# Heart Failure and Risk Assessment in Systemic Autoimmune Disease: Do Inflammation, Autoantibodies and Sex Matter?

**DOI:** 10.3390/biom16101406

**Published:** 2026-09-27

**Authors:** Brian B. Løgstrup, Inge Schjødt, Rikke Elmose Mols, Hans Erik Bøtker, Ellen-Margrethe Hauge

**Affiliations:** 1 Department of Cardiology, Aarhus University Hospital, 8200 Aarhus, Denmark; ingeschj@rm.dk; 2 Department of Clinical Medicine, Aarhus University, 8200 Aarhus, Denmark; boetker@clin.au.dk (H.E.B.); ellen.hauge@aarhus.rm.dk (E.-M.H.); 3 Department of Clinical Epidemiology, Aarhus University Hospital, 8200 Aarhus, Denmark; rikke.mols@clin.au.dk; 4 Department of Rheumatology, Aarhus University Hospital, 8200 Aarhus, Denmark

**Keywords:** heart failure, cardiovascular disease, systemic autoimmune rheumatic disease, inflammation, autoantibodies, sex, risk assessment

## Abstract

Heart failure (HF) is increasingly recognized as an important cardiovascular complication of systemic autoimmune rheumatic diseases (SARDs), which include rheumatoid arthritis, systemic lupus erythematosus, systemic sclerosis, Sjögren’s syndrome, and idiopathic inflammatory myopathies. Excess HF risk is only partly explained by conventional cardiovascular risk factors and ischemic heart disease. Chronic inflammation, endothelial and coronary microvascular dysfunction, immune-mediated myocardial injury, oxidative stress, fibrosis, and selected autoantibody-associated pathways may contribute to progressive myocardial dysfunction. Biological sex is also relevant because most SARDs predominantly affect women and sex influences immune regulation, vascular biology, myocardial remodeling, and HF phenotype. Current cardiovascular risk scores are primarily designed for atherosclerotic events and incompletely capture autoimmune disease-specific determinants of myocardial injury. This review summarizes HF across major SARDs and examines three interconnected modifiers of myocardial vulnerability—chronic inflammation, autoantibodies, and biological sex. We further discuss how disease activity and duration, biomarkers, global longitudinal strain, cardiovascular magnetic resonance, and conventional risk factors might be integrated into future risk assessment. A biology-centered approach may enable earlier recognition of subclinical myocardial involvement and individualized cardiovascular surveillance, but prospective validation is required before disease-specific HF-screening strategies can be recommended.

## 1. Introduction

Heart failure (HF) is a leading cause of morbidity and mortality worldwide, and its burden continues to increase with population aging and cardiometabolic disease [1,2]. Beyond established cardiovascular risk factors, chronic immune dysregulation is increasingly recognized as a contributor to myocardial dysfunction. Systemic autoimmune rheumatic diseases (SARDs)—including rheumatoid arthritis (RA), systemic lupus erythematosus (SLE), systemic sclerosis (SSc), Sjögren’s syndrome (SS), idiopathic inflammatory myopathies (IIM), and systemic vasculitides—combine persistent immune activation with vascular and, in some disorders, direct myocardial injury [3,4,5,6].

Cardiovascular research on SARDs has historically emphasized accelerated atherosclerosis. However, population-based studies demonstrate excess HF in RA and SLE, while myocardial fibrosis, inflammation, and ventricular dysfunction are increasingly recognized in SSc and IIM [7,8,9,10,11,12,13,14,15,16,17,18,19,20,21,22]. Both HF with preserved ejection fraction (HFpEF) and reduced ejection fraction (HFrEF) occur, suggesting that several mechanisms converge on different HF phenotypes [11,14,23,24].

Heart failure is a heterogeneous clinical syndrome that can be classified according to left ventricular ejection fraction (LVEF). The 2026 ESC Guidelines have simplified the previous LVEF-based classification into two principal phenotypes: HFrEF, defined as LVEF < 50%, and HFpEF, defined as LVEF ≥ 50%. The former category of heart failure with mildly reduced ejection fraction (HFmrEF; LVEF 41–49%) has therefore been incorporated into HFrEF, reflecting their similarities in pathophysiology and therapeutic response. HFpEF represents a heterogeneous syndrome in which symptoms and/or signs of HF occur despite preserved LVEF and requires objective evidence supporting cardiac dysfunction or elevated filling pressures [25]. This distinction is particularly relevant in systemic autoimmune diseases where inflammatory, microvascular, and fibrotic mechanisms may contribute to both HF phenotypes.

The pathways linking autoimmunity to HF extend beyond systemic inflammation. Cytokine signaling, oxidative stress, endothelial and coronary microvascular dysfunction, extracellular matrix remodeling, and fibrosis may be amplified by humoral immune responses and selected autoantibodies [4,26,27]. Biological sex may further modify these processes: many SARDs predominantly affect women, who exhibit distinct immune responses and are disproportionately affected by HFpEF [28,29,30,31,32,33].

**Figure 1:**
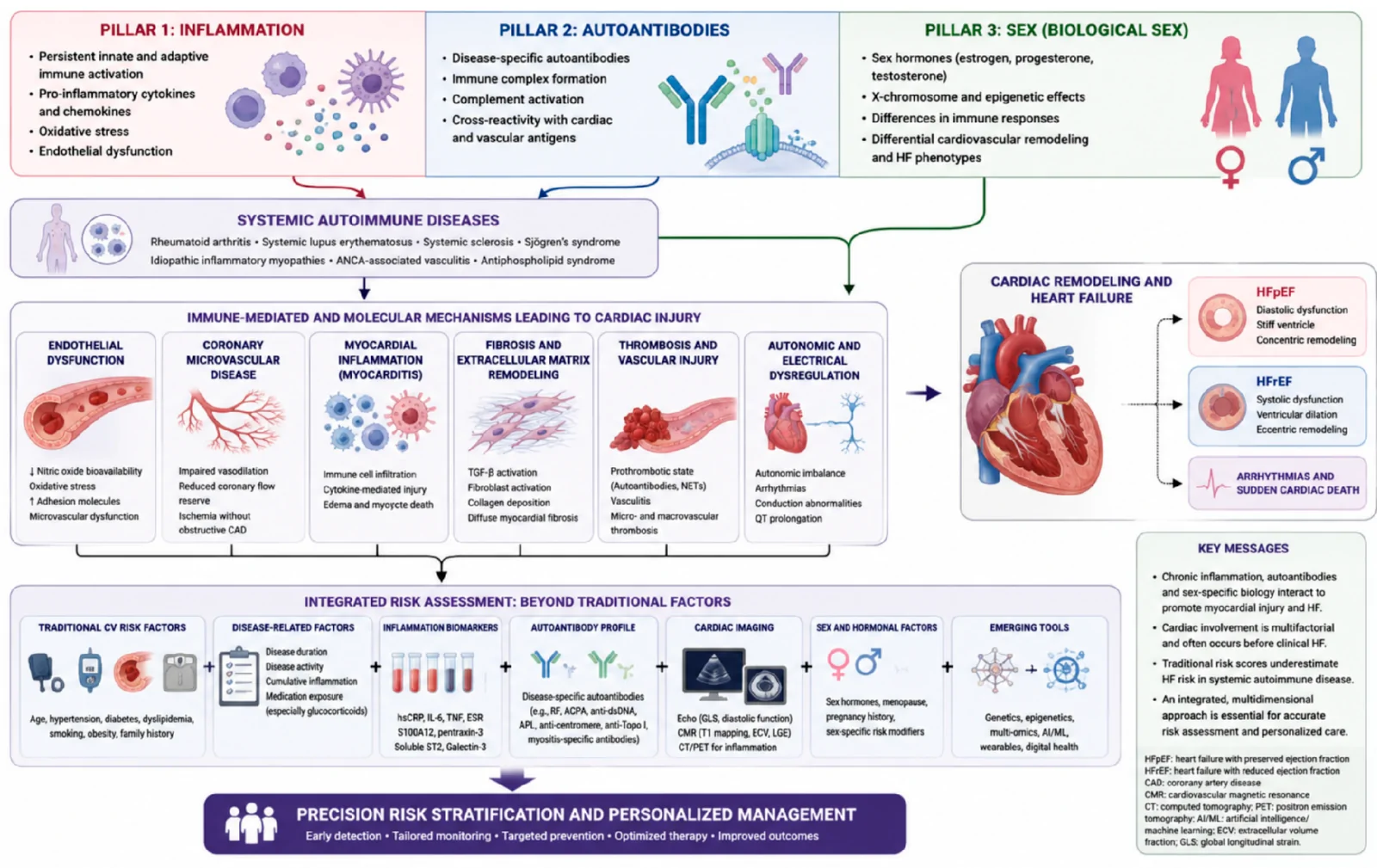
Conceptual framework: how sex, autoantibodies and inflammation drive heart failure in systemic autoimmune disease and inform a comprehensive risk assessment strategy.

Risk assessment remains challenging. SCORE2 estimates atherosclerotic cardiovascular risk and current EULAR recommendations emphasize conventional risk-factor management, whereas the recently developed SCORE2-HF predicts incident HF using conventional clinical and cardiometabolic variables [34,35,36]. None of these approaches specifically incorporates autoimmune disease activity, cumulative inflammatory burden, autoantibody profile, or subclinical myocardial abnormalities. This review therefore examines HF in SARDs through three interconnected biological domains—inflammation, autoantibodies, and sex—and considers how they may inform future HF risk assessment (Graphical Abstract/Figure 1).

## 2. Heart Failure Across Systemic Autoimmune Rheumatic Diseases

HF is an important but heterogeneous manifestation of SARDs. Myocardial dysfunction may arise independently of obstructive coronary artery disease through inflammation, microvascular dysfunction, fibrosis, pulmonary vascular disease, valvular disease, or myocarditis [3,4,5,6]. The relative contribution of these pathways differs by diagnosis and over time.

RA has the strongest epidemiological evidence. Population-based studies show increased risks of both ischemic and non-ischemic HF that are not fully explained by traditional risk factors [7,8,13]. Inflammatory burden is associated with subsequent HF, with recent data pointing towards HFpEF in particular [11,14,23]. Strain echocardiography and cardiac magnetic resonance imaging (CMRI) studies demonstrate subclinical systolic impairment, inflammation and diffuse fibrosis despite preserved LVEF [37,38].

SLE combines accelerated atherosclerosis with immune-mediated myocarditis, microvascular dysfunction, valvular disease, pulmonary hypertension, and antiphospholipid-associated thrombosis [9,15,16,39]. The high cardiovascular burden in relatively young patients illustrates the limitations of conventional risk assessment [36,40,41,42]. In SSc, primary myocardial disease is dominated by microvascular injury and fibrosis; strain imaging and CMRI can reveal abnormalities before conventional systolic dysfunction becomes evident [12,17,18,19,20]. In IIM, clinically overt cardiac disease is less common than subclinical involvement, but myocarditis, conduction disease, arrhythmias, and ventricular dysfunction are well described [10,21,22]. Evidence for primary SS and other systemic immune-mediated diseases is less extensive, although vascular inflammation, endothelial dysfunction, thrombosis, and shared inflammatory pathways plausibly increase cardiovascular risk [43,44,45].

Across diseases, two themes recur: myocardial injury may precede clinical HF, and cumulative immune activity may be more informative than a single inflammatory measurement. HFpEF is particularly relevant where systemic inflammation and microvascular dysfunction dominate, whereas extensive inflammatory injury or fibrosis may produce HFrEF [14,17,18,19,20,23,24]. Table 1 summarizes the principal disease-specific patterns.

## 3. Incidence and Clinical Presentation of Heart Failure Across SARDs

Epidemiological studies consistently demonstrate an increased incidence of HF across several SARDs, although absolute rates vary according to population, follow-up duration, age, and HF definition. In a Danish nationwide cohort, the incidence of hospitalized HF was 6.64 per 1000 person-years in patients with RA compared with 2.43 per 1000 person-years in the background population, corresponding to an adjusted incidence rate ratio of 1.30 [7]. In SLE, a Korean population-based study reported HF incidence rates of 3.11 versus 0.61 per 1000 person-years in patients with SLE and matched controls, respectively [46]. The relative and absolute burden appears particularly pronounced in SS: clinically evident HF occurred in 9.7% of patients within 10 years in a nationwide cohort, with an adjusted hazard ratio of 3.26 compared with individuals without SS [47]. Increased HF risk has also been demonstrated in primary Sjögren’s syndrome, with 10-year cumulative incidences of 4.0% versus 2.8% in matched controls, and in dermatomyositis/polymyositis, where corresponding 10-year risks were approximately 7.0% versus 4.6% [48]. These estimates should not be interpreted as direct comparisons between diseases because of important differences in study design and case ascertainment.

Importantly, HF or inflammatory cardiac involvement may occasionally precede recognition of the underlying SARD. This appears to be uncommon at the population level but is well documented in selected disease phenotypes. In a multicenter series of 29 patients with lupus myocarditis, myocarditis represented the first manifestation of SLE in 17 patients (59%), and two-thirds had an LVEF ≤ 45% at presentation [49]. HF as the presenting manifestation has also been reported in inflammatory myopathies, including polymyositis, and severe cardiac manifestations may occasionally precede or coincide with the diagnosis of systemic sclerosis [50]. Thus, otherwise unexplained HF—particularly when accompanied by myocarditis, pericardial disease, pulmonary vascular disease, systemic inflammation, autoantibodies, or other multisystem features—should prompt consideration of an underlying SARD.

## 4. Myocarditis as a Key Manifestation of Systemic Autoimmune Rheumatic Disease

Myocarditis represents an important form of non-ischemic cardiac involvement in SARDs. Its frequency, clinical presentation and underlying mechanisms differ between diseases, but it may contribute to acute HF, chronic ventricular dysfunction, conduction abnormalities and ventricular arrhythmias. Importantly, myocardial inflammation may be subclinical or occasionally precede recognition of the underlying autoimmune disease [39].

The mechanisms are heterogeneous and overlapping. Cellular immune infiltration, pro-inflammatory cytokines, oxidative stress, immune-complex and complement activation, autoantibody-mediated effects, endothelial dysfunction and coronary microvascular injury may all contribute to cardiomyocyte injury and subsequent fibrosis. The relative importance of these pathways differs between diseases [4,5,6,16,39]. In SLE, immune-mediated myocarditis may be severe and occasionally represents the first manifestation of disease [16,39,49]. In SSc, myocardial inflammation often coexists with microvascular dysfunction and progressive fibrosis, whereas in idiopathic inflammatory myopathies it may accompany systemic muscle inflammation and present with myocardial injury, arrhythmias or ventricular dysfunction [17,18,19,20,21,22]. In systemic vasculitides, additional mechanisms include small-vessel inflammation and, particularly in eosinophilic granulomatosis with polyangiitis, eosinophil-mediated myocardial injury [51].

An additional mechanism of particular relevance in immunosuppressed patients is infection-related myocardial inflammation. Immunosuppressive therapy used to control SARD may impair antiviral immune surveillance and facilitate reactivation or dissemination of latent viral infections, particularly members of the Herpesviridae family such as cytomegalovirus (CMV), Epstein–Barr virus and human herpesvirus-6 [52,53,54]. This is particularly relevant in patients receiving high-dose glucocorticoids or other potent immunosuppressive therapies. In SLE, for example, CMV disease and reactivation are more frequently observed in patients exposed to higher glucocorticoid doses and immunosuppressive agents, and rare cases of CMV-associated myocarditis have been reported [53,54]. Viral infection may therefore mimic, trigger or coexist with immune-mediated myocarditis, creating an important diagnostic and therapeutic challenge [52,55]. Distinguishing virus-associated from virus-negative immune-mediated myocarditis is clinically important because immunosuppressive therapy may be beneficial in the latter, whereas active viral infection should be excluded before initiating or intensifying immunosuppression in selected patients [52,55].

Diagnosis may be challenging because symptoms and biomarkers are non-specific and ventricular function may remain preserved in early disease. Assessment should therefore integrate clinical presentation, electrocardiogram, cardiac biomarkers, echocardiography and CMRI [39,56]. Contemporary ESC guidance emphasizes the central role of CMRI for non-invasive assessment of myocardial inflammation and fibrosis, while endomyocardial biopsy remains important in selected high-risk cases or when identification of a specific histological or etiological subtype may influence treatment [52].

Persistent or recurrent myocardial inflammation may promote fibrosis, adverse ventricular remodeling, chronic HF and an arrhythmogenic substrate [39]. Myocarditis may therefore represent an important intermediate phenotype linking systemic immune activation with subsequent HF and should be considered in patients with SARD who develop otherwise unexplained HF, troponin elevation, arrhythmias, conduction abnormalities or new ventricular dysfunction [39,52].

## 5. Why Does the Myocardium Become Injured?

**Figure 2:**
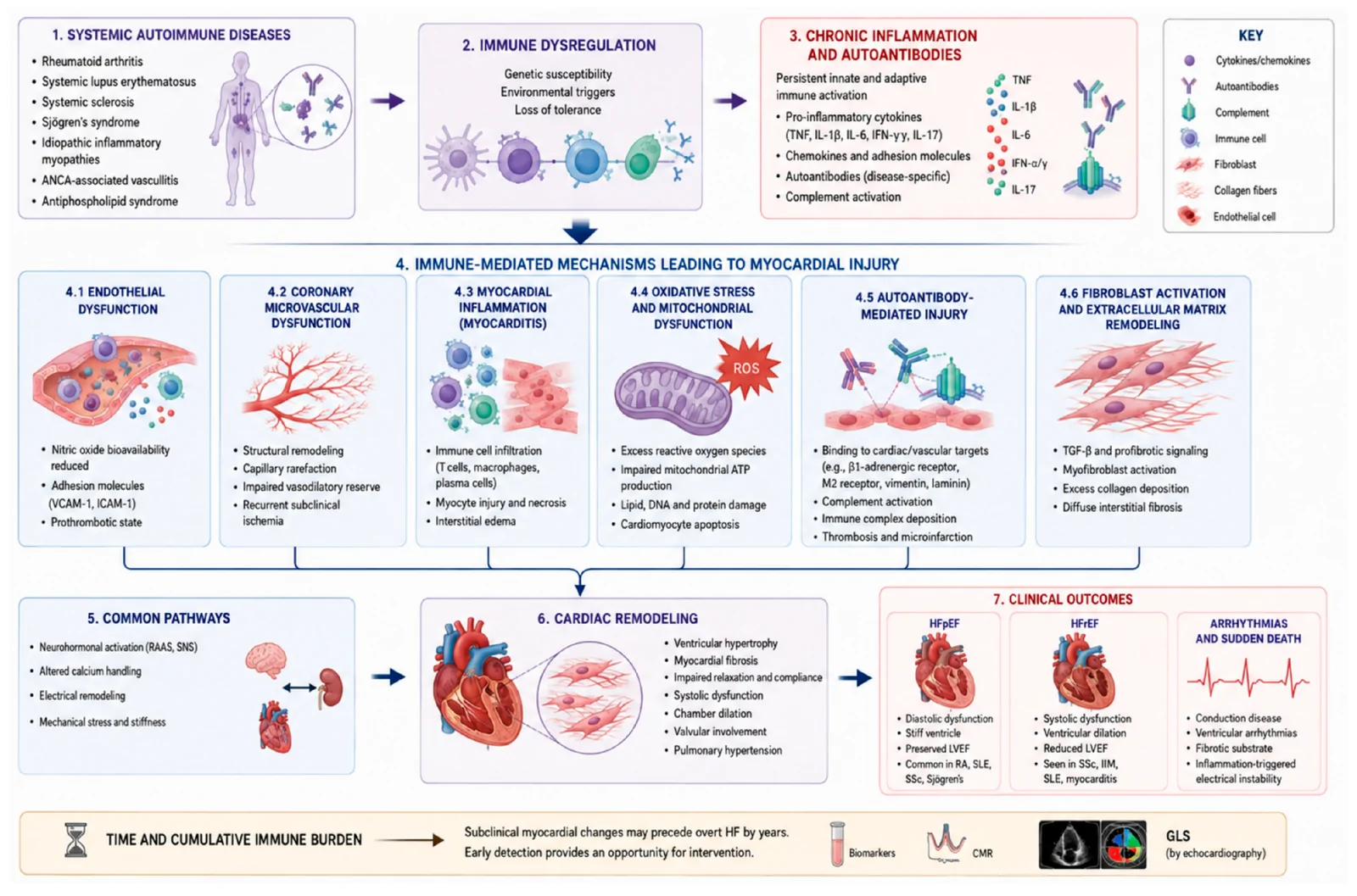
Pathophysiological cascade: From immune dysregulation to heart failure in systemic autoimmune diseases.

Cardiac involvement in SARDs is multifactorial (Figure 2). Chronic inflammation, immune dysregulation, endothelial and coronary microvascular dysfunction, direct myocardial inflammation, oxidative stress, and fibrosis frequently coexist and can cause progressive injury without obstructive coronary artery disease [4,6,26,37]. Sensitive imaging may detect abnormalities before overt ventricular dysfunction, supporting a continuum from subclinical immune-mediated injury to symptomatic HF [37,38,56].

Persistent activation of innate and adaptive immunity exposes the myocardium and vasculature to pro-inflammatory mediators, including TNF-alpha, IL-1, IL-6, interferons, and related pathways. These signals impair endothelial function, increase oxidative stress, alter cardiomyocyte biology, and activate profibrotic remodeling [4,26]. In RA, chronic and cumulative inflammatory activity is associated with HF risk, particularly HFpEF [14,23].

The endothelium is a key interface between systemic inflammation and the heart. Reduced nitric oxide bioavailability and endothelial activation impair vasodilatory and antithrombotic function, while coronary microvascular dysfunction may reduce myocardial perfusion despite angiographically normal epicardial arteries [4,6,26]. Repeated microvascular ischemic stress can promote impaired relaxation and fibrosis, fitting the inflammatory-microvascular paradigm of HFpEF [24].

Recent ESC guidelines on cardiomyopathies and inflammatory myocardial and pericardial syndromes further emphasize that myocardial disease should be approached using systematic phenotypic and etiological characterization rather than ventricular function alone [52,57]. Multimodality imaging, particularly CMRI, plays a central role in identifying myocardial inflammation, fibrosis and other tissue abnormalities, while endomyocardial biopsy may be considered in selected patients when identification of the underlying inflammatory or immune-mediated process is clinically relevant. These concepts are particularly pertinent to SARDs, in which myocardial involvement may be subtle, heterogeneous and present before overt HF develops.

Direct myocardial inflammation is particularly relevant in SLE, IIM, SSc, and systemic vasculitis. Although clinically overt myocarditis is uncommon, CMRI can identify edema, inflammation, focal scar, and diffuse tissue abnormalities in some patients [17,18,19,20,21,22,39,56]. Persistent injury may lead to fibroblast activation and extracellular matrix expansion. Diffuse fibrosis increases ventricular stiffness and may eventually impair systolic function while providing an arrhythmogenic substrate [12,17,18,19,20,37].

These mechanisms are interdependent rather than sequential. Inflammation promotes endothelial dysfunction and oxidative injury; microvascular disease and myocarditis promote fibrosis; and humoral immune responses may amplify vascular or myocardial damage. The dominant combination likely determines whether the clinical phenotype is predominantly HFpEF, HFrEF, arrhythmia, or mixed disease [4,6,23,24].

## 6. Autoantibodies: Beyond Disease Classification—Potential Mediators of Myocardial Injury

Autoantibodies define many SARDs and are indispensable for diagnosis and phenotyping. Their cardiovascular relevance, however, ranges from well-established pathogenic effects to associations that may simply mark a particular disease endotype. This distinction is important: for most autoantibodies, direct causality for myocardial dysfunction has not been demonstrated [27].

Autoantibody levels may also change over time, although their longitudinal behavior differs substantially between antibody specificities and diseases [58]. In SLE, anti-dsDNA titres may fluctuate with disease activity and, in some patients, increases precede clinical flares, although the association is not sufficiently consistent to use serial titres in isolation for clinical decision-making. In contrast, several disease-defining autoantibodies, particularly anticentromere and anti-topoisomerase I antibodies in SSc, tend to remain relatively stable over time and primarily provide diagnostic and phenotypic information rather than reflecting short-term disease activity [59]. Consequently, the clinical interpretation of autoantibodies should consider temporal changes, disease context and assay methodology, and a single measurement may not fully capture the dynamic humoral immune response.

Several non-exclusive mechanisms are biologically plausible. Immune complexes can activate complement and recruit inflammatory cells; antibodies directed against endothelial or phospholipid-associated targets can promote endothelial activation and thrombosis; and experimental cardiac autoantibodies may alter receptor signaling or cardiomyocyte function [27]. The strongest clinical example is antiphospholipid antibodies, where thrombosis and vascular injury can cause macrovascular or microvascular myocardial damage [44].

Beyond disease-specific autoantibodies, cardiac-specific autoantibodies deserve particular consideration in the context of myocarditis. Organ-specific anti-heart autoantibodies (AHAs) and anti-intercalated disk autoantibodies (AIDAs) have been identified as markers of cardiac autoimmunity, and contemporary ESC guidance recognizes their potential relevance in selected patients with suspected immune-mediated myocarditis [52,60]. Cardiac myosin represents an important myocardial autoantigen, and anti-cardiac myosin antibodies have been demonstrated in patients with myocarditis and dilated cardiomyopathy [60,61]. Experimental and translational studies suggest that immune responses against cardiac myosin may contribute to the initiation or perpetuation of myocardial inflammation, while anti-cardiac myosin antibodies may exert functional effects through cross-reactivity with β-adrenergic receptors [27,61]. Functional autoantibodies directed against the β1-adrenergic receptor may consequently alter cardiomyocyte signaling and contribute to myocardial dysfunction, arrhythmias and adverse remodeling [27,61]. Importantly, however, the presence of cardiac autoantibodies does not by itself establish direct antibody-mediated causality in an individual patient, and their pathogenic relevance may differ according to antibody specificity and disease stage [27,52]. Cardio-specific antibodies, including AHAs and AIDAs, may therefore provide additional evidence of an autoimmune myocardial process in selected patients, although routine clinical application remains limited by assay availability and incomplete standardization [52].

In RA, rheumatoid factor and anti-citrullinated protein antibodies (ACPAs) identify seropositive disease and often track a more inflammatory phenotype. Associations with cardiovascular disease have been reported, but whether ACPAs directly drive myocardial remodeling remains uncertain [26,27]. In SLE, anti-dsDNA antibodies participate in immune-complex biology, antiphospholipid antibodies confer thrombotic risk, and anti-Ro/SSA is firmly linked to congenital conduction disease; evidence for direct adult myocardial injury is much less definitive [16,27,44].

In SSc, anti-topoisomerase I, anticentromere, and anti-RNA polymerase III antibodies primarily define clinical subsets and organ-risk profiles rather than established direct cardiac toxicity [12,17,18,19,20]. Similarly, myositis-specific antibodies identify distinct IIM phenotypes, but robust prospective data linking individual antibodies to incident HF remain limited [10,21,22]. Anti-Ro/SSA and anti-La/SSB dominate the serology of primary SS; current evidence is insufficient to use these antibodies alone for HF prediction [43].

Accordingly, autoantibodies are best considered potential markers of biological endotypes rather than stand-alone cardiovascular biomarkers (Table 2). Their future value may lie in combination with cumulative inflammatory burden, cardiac biomarkers, and imaging. Prospective studies with serial serology and standardized cardiovascular phenotyping are required to establish whether antibody signatures add incremental predictive value [27,42].

## 7. Sex Matters: Biological Sex as a Determinant of Heart Failure Risk in Systemic Autoimmune Rheumatic Diseases

Biological sex is a major determinant of both autoimmune susceptibility and cardiovascular physiology. Many SARDs predominantly affect women, reflecting differences in sex hormones, X-chromosome biology, epigenetic regulation, and innate and adaptive immunity [28,29,30]. These mechanisms influence immune tolerance and autoantibody production and may also modify vascular and myocardial responses to chronic inflammation.

Estrogens and androgens have context-dependent effects on immune cells, endothelial function, oxidative stress, and remodeling. X-linked immune genes and incomplete X-chromosome inactivation may further contribute to stronger immune responses in women [28,29,30]. Cardiovascular structure and HF phenotype also differ by sex; HFpEF is more frequent in women, whereas HFrEF is more frequent in men [32,33].

This overlap is potentially important in SARDs. Systemic inflammation, endothelial dysfunction, coronary microvascular impairment, and diffuse fibrosis are central features of contemporary HFpEF models and are also common in autoimmune disease [4,23,24,26]. Thus, autoimmune inflammation may amplify pathways by which women are already disproportionately affected. This is a biologically plausible framework, but direct prospective evidence that female sex specifically mediates autoimmune-associated HFpEF remains limited.

Menopause may represent an additional modifier. Loss of ovarian hormone exposure is accompanied by adverse changes in vascular function, arterial stiffness, body composition, and cardiometabolic risk [31]. In women with longstanding SARDs, these changes may interact with cumulative inflammatory exposure. However, menopausal status and reproductive history are rarely incorporated into autoimmune cardiovascular cohorts, and evidence is currently insufficient to recommend menopause-specific cardiac screening.

Future studies should therefore treat sex as more than a statistical covariate while avoiding overinterpretation. Sex-disaggregated analyses, menopausal status, reproductive history, and sex-by-inflammation interactions may help clarify why HF phenotype and trajectory differ among patients with SARDs [28,29,30,31,32,33].

## 8. Risk Assessment: Towards an Integrated Cardiovascular Risk Model for Systemic Autoimmune Rheumatic Diseases

Conventional cardiovascular risk assessment incompletely addresses HF in SARDs. SCORE2 estimates fatal and non-fatal atherosclerotic cardiovascular disease, while EULAR recommendations emphasize management of conventional risk factors in rheumatic diseases [34,44]. SCORE2-HF is an important advance because it directly estimates incident HF, but it was developed from conventional clinical and cardiometabolic variables rather than autoimmune-specific determinants [35].

This limitation matters because traditional risk factors do not fully explain cardiovascular risk in SLE and RA [23,36,40,41,42,45]. Disease duration, cumulative inflammatory burden, renal and other organ involvement, treatment exposure, and autoantibody-defined phenotype may modify risk. Nevertheless, evidence is strongest for some diseases—notably RA and SLE—and no integrated SARD-specific HF model has yet been prospectively validated.

Blood versus imaging biomarkers: complementary tools for detecting myocardial involvement.

Blood and imaging biomarkers provide complementary information on cardiovascular involvement in SARDs. Circulating biomarkers have the advantages of accessibility, reproducibility, relatively low cost, and suitability for repeated assessment. Natriuretic peptides, particularly BNP and NT-proBNP, primarily reflect myocardial wall stress and hemodynamic burden, whereas high-sensitivity cardiac troponins reflect cardiomyocyte injury. Both may therefore identify clinically relevant cardiac involvement and provide prognostic information. However, neither biomarker is specific for the underlying myocardial process. Natriuretic peptide concentrations are influenced by age, renal function, atrial fibrillation and obesity, while troponin elevation may occur in renal dysfunction, pulmonary hypertension, systemic illness and other causes of myocardial injury. Interpretation of cardiac troponin may be particularly challenging in SARDs because heterophile antibodies, rheumatoid factor and macro-troponin complexes can occasionally interfere with immunoassays [62,63]. Thus, circulating biomarkers are well suited to initial risk stratification and longitudinal surveillance, but abnormal values generally require further phenotypic characterization.

Emerging proteomic approaches may extend conventional blood-based assessment by identifying biological pathways related to inflammation, fibrosis, vascular dysfunction and hemodynamic stress [64,65]. In the RA-HF study, broad cardiovascular proteomic profiling identified biomarker signatures associated with congestion, inflammation, fibrosis and renin–angiotensin system activation in patients with RA and HF [65]. However, circulating biomarkers provide limited information regarding the anatomical location or mechanism of myocardial injury, and their ability to identify early, localized or diffuse subclinical cardiac involvement remains uncertain. Indeed, studies in SSc have demonstrated only modest concordance between troponin or natriuretic peptides and abnormalities detected by CMRI, supporting a multimodal rather than biomarker-only diagnostic approach [62].

Imaging biomarkers address several of these limitations by providing direct assessment of cardiac structure, function and, increasingly, tissue composition. Conventional echocardiography remains the first-line imaging modality because it provides information on ventricular systolic and diastolic function, chamber geometry, valvular disease and pulmonary pressures. Advanced echocardiographic techniques, particularly global longitudinal strain (GLS), may identify subtle myocardial dysfunction despite preserved LVEF. In RA, impaired left- and right ventricular longitudinal strain has been demonstrated in patients without recognized cardiovascular disease and has been associated with indices of disease severity [38]. More recent prospective data similarly suggest that GLS can identify a subgroup with subclinical LV dysfunction despite preserved LVEF, illustrating the potential value of deformation imaging as an intermediate phenotype between systemic inflammation and overt HF [66].

CMRI provides an additional dimension because it combines accurate assessment of ventricular volumes and function with myocardial tissue characterization. Late gadolinium enhancement identifies focal replacement fibrosis, while native T1 mapping and extracellular volume fraction may detect diffuse interstitial expansion and fibrosis that are not apparent from LVEF or conventional imaging. T2-based techniques provide information regarding myocardial edema and active inflammation [37,67]. In RA, CMRI studies have demonstrated increased native T1, extracellular volume and focal myocardial fibrosis despite preserved conventional ventricular function, with tissue abnormalities associated with impaired myocardial strain and disease activity [37]. Similar observations have been made in SSc, where multiparametric CMRI may identify diffuse fibrosis or inflammation before overt ventricular dysfunction develops. The European Association of Cardiovascular Imaging therefore recognizes CMRI as an important modality for evaluation of suspected cardiac involvement in SARDs, particularly when conventional assessment does not adequately explain symptoms or biomarker abnormalities [56].

The major clinical advantage of imaging biomarkers is therefore not simply increased sensitivity but the ability to phenotype the underlying cardiac process. In a patient with an SAD, elevated NT-proBNP or troponin may indicate that cardiac involvement is present, whereas echocardiography and CMRI can help distinguish ventricular dysfunction, abnormal filling pressures, pulmonary hypertension, myocarditis, microvascular disease and focal or diffuse myocardial fibrosis. Imaging may consequently refine diagnosis in patients in whom symptoms and circulating biomarkers are non-specific or discordant. Nevertheless, abnormal imaging biomarkers should not automatically be equated with clinically relevant HF. Many studies remain small and cross-sectional, disease-specific reference values are incompletely established, and evidence that systematic imaging of asymptomatic patients improves clinical outcomes is lacking [37,56,62]. Accordingly, the most pragmatic strategy may be a stepwise multimodal approach, in which clinical phenotype and circulating biomarkers identify patients requiring more detailed characterization by echocardiography, GLS and, in selected cases, CMRI [17,18,19,20,37,38,56]. Such integration of molecular and imaging biomarkers may facilitate earlier and more precise identification of immune-mediated myocardial involvement while avoiding indiscriminate cardiovascular imaging.

A pragmatic future framework could therefore combine (1) conventional cardiovascular and HF risk factors; (2) autoimmune disease characteristics, particularly cumulative inflammatory activity and organ involvement; (3) serological phenotype where clinically informative; (4) cardiac biomarkers; (5) targeted global longitudinal strain by echocardiography or CMRI imaging; and (6) sex-specific modifiers. Such a model should be tested for incremental discrimination, calibration, clinical utility, and cost-effectiveness before implementation [35,42,44].

## 9. Therapeutic Considerations: Integrating Antirheumatic and Heart Failure Treatment

Management of HF in SADs requires simultaneous consideration of the cardiac phenotype and the underlying inflammatory disease. In the absence of randomized trials specifically targeting autoimmune-associated HF, patients with established HF should generally receive guideline-directed HF therapy according to contemporary recommendations, irrespective of the presence of an autoimmune disease [25,44]. The 2026 ESC HF Guidelines emphasize phenotype-directed treatment across the spectrum of HF, including early implementation of evidence-based pharmacological therapy and appropriate management of congestion and cardiovascular comorbidities. Treatment should nevertheless be individualized according to renal function, blood pressure, electrolyte disturbances, frailty, and potential interactions with immunomodulatory therapy.

Optimal control of the underlying rheumatic disease represents a complementary therapeutic goal. Persistent systemic inflammation is associated with cardiovascular and HF risk, particularly in RA, but there is currently insufficient evidence that any individual disease-modifying antirheumatic drug (DMARD) should be selected specifically to prevent HF [68]. Accordingly, antirheumatic treatment should primarily follow disease-specific treat-to-target principles, while cardiovascular comorbidity is incorporated into drug selection. EULAR recommendations emphasize control of disease activity and minimization of chronic glucocorticoid exposure, particularly because prolonged glucocorticoid treatment may aggravate hypertension, diabetes, obesity and other cardiovascular risk factors [44].

Several commonly used antirheumatic treatments warrant particular attention in patients with HF. The principal therapeutic considerations for patients with SARD and concomitant cardiac involvement are summarized in Table 3.

Non-steroidal anti-inflammatory drugs may cause sodium and fluid retention, impair renal function and precipitate HF decompensation and should therefore be avoided or used cautiously in established HF. TNF-α inhibition represents another important consideration. In patients with RA and New York Heart Association class III–IV HF, the American College of Rheumatology conditionally recommends a non-TNF biologic or targeted synthetic DMARD rather than a TNF inhibitor and recommends switching away from TNF inhibition if HF develops [68,69]. These recommendations are based largely on trials of TNF inhibition in patients with advanced HF rather than RA-specific randomized evidence and are therefore supported by low-certainty evidence [70].

A different approach may be required when cardiac dysfunction reflects active immune-mediated myocardial disease rather than conventional HF alone. Myocarditis, pericardial inflammation and inflammatory myocardial involvement may require disease-specific immunosuppressive therapy in addition to standard cardiac treatment. This is particularly relevant in systemic sclerosis, SLE and inflammatory myopathies, although the evidence is predominantly observational and therapeutic strategies should be individualized according to the underlying disease and evidence of active myocardial inflammation. In SSc, for example, corticosteroids and conventional immunosuppressive agents have been used in clinically suspected or documented myocarditis, but robust randomized evidence demonstrating improvement in cardiac outcomes remains lacking [71].

Thus, cardiac and antirheumatic therapies should not be regarded as competing strategies. A multidisciplinary approach integrating guideline-directed HF therapy, adequate control of systemic inflammation, avoidance of potentially HF-aggravating medications, and targeted immunosuppression when active myocardial inflammation is demonstrated provides the most rational current management strategy. Prospective studies are needed to determine whether specific anti-inflammatory or immunomodulatory approaches can modify the development or progression of HF in SARDs.

## 10. Future Directions: From Immune-Mediated Cardiac Injury to Precision Cardiovascular Medicine

The next phase of cardio-immunology/rheumatology should move from documenting excess cardiovascular risk toward identifying which biological pathways predict progressive myocardial disease. Large prospective cohorts should combine repeated measures of disease activity, treatment exposure, autoantibodies, cardiac biomarkers, echocardiography, CMRI imaging, and adjudicated HF phenotypes. Serial assessment is essential because autoimmune activity and myocardial injury are dynamic [5,6,23,42].

A second priority is biological phenotyping across diagnostic boundaries. Patients with different SARDs may share inflammatory, microvascular, fibrotic, or antibody-associated pathways, whereas patients with the same diagnosis may have very different myocardial risk. Multi-omics and systems-biology approaches could help define such endotypes, but they should complement—not replace—careful clinical and imaging phenotyping [64,65,72].

Finally, clinical utility must be demonstrated. Randomized or strategy trials are needed to determine whether risk-adapted imaging, biomarker-guided surveillance, intensified control of inflammation, or earlier cardioprotective therapy prevents progression to symptomatic HF. Until such evidence exists, prevention should focus on rigorous management of established cardiovascular risk factors, optimal control of autoimmune disease, and targeted cardiac evaluation when symptoms, biomarkers, or disease phenotype raise suspicion [44].

## 11. Conclusions

HF is an important and probably underrecognized cardiovascular complication of SARDs. Excess risk cannot be attributed to accelerated atherosclerosis alone. Chronic inflammation, endothelial and coronary microvascular dysfunction, myocardial inflammation, oxidative injury, and fibrosis provide interconnected pathways from systemic autoimmunity to adverse cardiac remodeling.

Three modifiers deserve particular attention. Cumulative inflammation is consistently linked to myocardial risk, especially in RA; selected autoantibodies may identify pathogenic or prognostic endotypes, although direct causality is established for only a minority; and biological sex influences immune regulation, vascular biology, and HF phenotype.

Current risk models do not capture this biological complexity. SCORE2-HF provides a contemporary framework for HF prediction in the general population, but autoimmune-specific risk assessment will require prospective validation of disease activity, serology, biomarkers, and imaging markers such as global longitudinal strain by echocardiography and CMRI imaging. The goal should be earlier identification of clinically meaningful myocardial involvement without indiscriminate screening. A biology-centered, multidisciplinary approach offers a rational path toward more precise prevention of HF in systemic autoimmunity.

## Figures and Tables

**Table 1:** Heart failure in systemic autoimmune disease.

Disease	Relative HF Risk	Predominant HF Phenotype	Dominant Mechanisms	Typical Imaging Findings
Rheumatoid arthritis	↑↑	HFpEF > HFrEF	Chronic inflammation, CMD, endothelial dysfunction	Reduced GLS, diastolic dysfunction
Systemic lupus erythematosus	↑↑↑	HFpEF/HFrEF	Myocarditis, APS, microvascular disease, inflammation	GLS impairment, CMR inflammation
Systemic sclerosis	↑↑	HFpEF → HFrEF	Diffuse fibrosis, microvascular ischemia	Increased ECV, LGE, impaired GLS
Sjögren’s syndrome	↑	Mainly HFpEF	Endothelial dysfunction, chronic inflammation	Mild GLS reduction
Idiopathic inflammatory myopathies	↑↑	HFrEF > HFpEF	Myocarditis, fibrosis	CMR edema/LGE, reduced LVEF
ANCA-associated vasculitis	↑	Variable	Vasculitis, inflammation	Variable
Antiphospholipid syndrome	↑	Variable	Thrombosis, microvascular ischemia	Ischemic and non-ischemic findings

Abbreviations: HF: heart failure; HFpEF: heart failure with preserved ejection fraction; HFrEF: heart failure with reduced ejection fraction; CMD: coronary microvascular dysfunction; GLS: global longitudinal strain; CMR: cardiac magnetic resonance; ECV: extracellular volume; LGE: late gadolinium enhancement.

**Table 2:** Autoantibodies and cardiac mechanisms.

Autoantibody	Disease(s)	Proposed Cardiac Mechanism	Clinical Evidence	Potential HF Relevance
Rheumatoid factor (RF)	RA	Marker of systemic inflammation; vascular injury	Moderate	Indirect ↑ HF risk
Anti-citrullinated protein antibodies (ACPA)	RA	Endothelial dysfunction, vascular inflammation, myocardial citrullination (proposed)	Moderate	HFpEF/remodeling
Anti-dsDNA	SLE	Immune complexes, complement activation, myocarditis	Strong	HF, myocarditis
Anti-Ro/SSA	SLE, Sjögren’s	Conduction abnormalities, myocardial inflammation	Moderate	Arrhythmias, HF
Anti-La/SSB	SLE, Sjögren’s	Immune-mediated tissue injury	Limited	Possible HF association
Antiphospholipid antibodies	APS, SLE	Thrombosis, endothelial activation, microvascular ischemia	Strong	Ischemic and non-ischemic HF
Anti-topoisomerase I (Scl-70)	Systemic sclerosis	Fibrosis-associated phenotype	Moderate	Fibrotic remodeling
Anti-centromere	Systemic sclerosis	Microvascular dysfunction	Moderate	HFpEF/pulmonary vascular disease
Anti-Jo-1	Inflammatory myopathies	Immune-mediated myocarditis	Moderate	Inflammatory cardiomyopathy
Anti-MDA5	Inflammatory myopathies	Severe inflammatory phenotype	Emerging	Possible myocarditis/HF
β1-adrenergic receptor antibodies	Multiple (experimental)	Direct cardiomyocyte signaling dysfunction	Emerging	Dilated cardiomyopathy/HFrEF

Abbreviations: HF: heart failure; RA: rheumatoid arthritis; HFpEF: heart failure with preserved ejection fraction; SLE: systemic lupus erythematosus; HFrEF: heart failure with reduced ejection fraction.

**Table 3:** Main therapeutic considerations in systemic autoimmune disease with cardiac involvement or heart failure.

Clinical Issue	Main Therapeutic Consideration	Key Points
Active systemic autoimmune disease	Optimize disease-modifying antirheumatic therapy according to disease-specific recommendations	Persistent inflammatory activity may contribute to cardiovascular and myocardial injury. Treatment should follow established rheumatological treat-to-target principles; however, there is currently insufficient evidence to select a specific DMARD solely for prevention of HF.
Established HFrEF or HFpEF	Treat according to contemporary HF guidelines	Patients with systemic autoimmune disease and established HF should generally receive guideline-directed HF therapy according to cardiac phenotype, with individualization according to renal function, blood pressure, electrolytes, frailty and concomitant immunomodulatory treatment. The 2026 ESC Guidelines recommend phenotype-directed treatment and updated evidence-based therapies across the LVEF spectrum.
Glucocorticoid exposure	Use the lowest effective dose for the shortest feasible duration	Chronic glucocorticoid exposure may adversely affect blood pressure, glucose metabolism, body composition and overall cardiovascular risk. EULAR recommendations emphasize limiting exposure and discontinuing treatment when clinically feasible.
NSAID treatment	Avoid or use cautiously in patients with established HF	NSAIDs may promote sodium and fluid retention and worsen renal function, thereby contributing to HF decompensation. Their necessity and duration should therefore be carefully evaluated in patients with cardiac dysfunction.
TNF-α inhibitors	Exercise particular caution in advanced symptomatic HF	In RA patients with NYHA class III–IV HF, ACR conditionally recommends a non-TNF biologic or targeted synthetic DMARD rather than a TNF inhibitor. If HF develops during TNF inhibition, switching to a non-TNF agent is conditionally recommended. These recommendations are based on very-low-certainty evidence.
Suspected immune-mediated myocarditis or inflammatory myocardial involvement	Combine cardiac treatment with disease-specific immunomodulation when active inflammation is demonstrated	Myocarditis or inflammatory myocardial disease may require immunosuppressive treatment in addition to conventional HF management. Evidence is strongest for selected diseases such as systemic sclerosis, SLE and inflammatory myopathies, but remains predominantly observational.
Pericardial, pulmonary vascular or other disease-specific cardiac involvement	Treat according to the dominant pathophysiological phenotype	Cardiac manifestations should be phenotyped before treatment because management of pericarditis, pulmonary hypertension, myocarditis, valvular disease or conventional ventricular HF differs substantially.
Multidisciplinary management	Integrate cardiology and rheumatology expertise	Optimal management requires simultaneous assessment of HF phenotype, autoimmune disease activity, treatment-related cardiovascular effects and relevant comorbidities.

Abbreviations: DMARD, disease-modifying antirheumatic drug; HF, heart failure; HFpEF, heart failure with preserved ejection fraction; HFrEF, heart failure with reduced ejection fraction; NSAID, non-steroidal anti-inflammatory drug; NYHA, New York Heart Association; RA, rheumatoid arthritis; SLE, systemic lupus erythematosus.

## Data Availability

No new data were created or analyzed in this study. Data sharing is not applicable to this article.

## Abbreviations

HF	Heart failure
SARDs	Systemic autoimmune rheumatic diseases
RA	Rheumatoid arthritis
SLE	Systemic lupus erythematosus
SSc	Systemic sclerosis
SS	Sjögren’s syndrome
IIM	Idiopathic inflammatory myopathies
HFpEF	Heart failure with preserved ejection fraction
HFrEF	Heart failure with reduced ejection fraction
LVEF	Left ventricular ejection fraction
HFmrEF	Heart failure with mildly reduced ejection fraction
CMRI	Cardiac magnetic resonance imaging
CMV	Cytomegalovirus
TNF-alpha	Tumor necrosis factor alpha
IL	Interleukin
AHAs	Anti-heart autoantibodies
AIDAs	Anti-intercalated disc autoantibodies
ACPAs	Anti-citrullinated protein antibodies
GLS	Global longitudinal systolic strain
DMARD	Disease-Modifying Antirheumatic Drug
